# A Clinicoimmunohistopathologic Study of Anetoderma: Is Protruding Type More Advanced in Stage Than Indented Type?

**DOI:** 10.1155/2016/4325463

**Published:** 2016-12-28

**Authors:** Jung Eun Kim, Ki Min Sohn, Young Jun Woo, Kwan Ho Jeong, Miri Kim, Jeong Deuk Lee, Jun Young Lee, Hyun Jeong Park, Gyong Moon Kim, Chul Jong Park, Dong Soo Yu, Hoon Kang

**Affiliations:** Department of Dermatology, College of Medicine, The Catholic University of Korea, Seoul, Republic of Korea

## Abstract

*Background*. The clinical and histopathologic classification of anetoderma are not well characterized.* Objective*. We aimed to investigate the clinical and histopathologic characteristics of anetoderma and to correlate clinical phenotypes with immunohistopathologic findings.* Methods*. We retrospectively reviewed the medical records of 30 patients with anetoderma and performed immunohistochemistry for elastin, fibrillin-1, metalloproteinase- (MMP-) 2, MMP-7, MMP-9, and MMP-12, and tissue inhibitor of metalloproteinase- (TIMP-) 1 and TIMP-2.* Results*. Protruding type (*n* = 17) had a longer disease duration and more severe loss of elastin, without changes in fibrillin, than indented type (*n* = 13). MMP-2 and MMP-9 showed significantly higher expressions in the dermis compared with controls (*p* < 0.05). MMP-7 and MMP-12 showed little expressions in both anetoderma and control tissue. TIMP-1 was highly expressed in anetoderma lesions and controls. TIMP-2 expression was variable.* Conclusions*. Our findings suggest that protruding type anetoderma may represent a more advanced stage and that MMP-2 and MMP-9 could be responsible for elastic fiber degradation in anetoderma.

## 1. Introduction

Anetoderma is a skin disorder characterized by focal loss of dermal elastic tissue. Clinically, it can present as various types of flaccid skin, such as protruding (raised), indented (depressed), or flat [1]. Anetoderma is classified as primary when it occurs idiopathically from normal skin and secondary when it is preceded by an inflammatory or tumor-related skin condition, such as varicella, lupus erythematosus, lichen planus, or pilomatricoma [2–5]. Primary and secondary anetoderma have both been reported to be associated with autoimmune disorders, including Grave's disease, autoimmune hemolysis, systemic sclerosis, Hashimoto's thyroiditis, and lupus erythematosus [6–9]. Some patients with anetoderma also present with antiphospholipid syndrome, and histologic specimens from these patients reveal complement and immunoglobulin deposit around and microthrombi within blood vessels [3, 10, 11].

Histopathologically, anetoderma is typically subclassified as Jodassohn-Pellizzari type (inflammatory type) or Schweninger-Buzzi type (noninflammatory type). However, both pathologic phenotypes are simultaneously observed in some patients with anetoderma [12], and the clinical courses of these two pathologic subtypes do not differ [13]. To date, there is no proper clinical or histological classification of anetoderma, and the etiology of this disease remains unclear.

Whether the decrease and change in elastic fiber content in anetoderma arises from decreased production or increased destruction is not well understood. An imbalance in levels of the matrix metalloproteinases (MMPs) and tissue inhibitor of metalloproteinases (TIMPs) has been suggested as one explanation of the pathophysiology of anetoderma; however, a definitive immunohistopathologic analysis of these proteins in a large cohort of anetoderma patients has not yet been performed [14, 15]. Limited studies suggest that immunologic mechanisms may play a role in this elastolytic process [7]. Elastic fibers consist of elastin and microfibrils, both of which are possible targets of autoantibodies; however, the precise target antigen or autoantibody has not yet been identified.

There has been little evidence delineating exactly how anetoderma develops and why similar histopathologic features can present with different clinical features, such as protruding or indented skin phenotypes. We aimed to investigate the clinical characteristics of anetoderma patients and to correlate these findings with immunohistochemical changes in the MMPs that most effectively degrade elastic tissue (MMP-2, MMP-7, MMP-9, and MMP-12) and their physiologic inhibitors (TIMP-1 and TIMP-2) [14, 16]. To the best of our knowledge, this is the first study to make these clinical and histopathologic observations in patients with anetoderma.

## 2. Methods

This study was approved by the institutional review board of The Catholic University of Korea (XC13RIMI0123) and all subjects gave informed consent. A total of 30 subjects with anetoderma were enrolled in this study between January 1, 2003, and December 31, 2012. All patients were diagnosed with anetoderma from biopsy specimens. To exclude selection bias, all patients diagnosed with anetoderma were enrolled in this study. Medical records were retrospectively reviewed for information on age, sex, disease duration, lesion topography, lesion number, and antecedent inflammatory events. Biopsy specimens of anetoderma lesions were used for hematoxylin and eosin and Verhoeff-Van Gieson staining.

### 2.1. Immunohistochemistry

Biopsy specimens for immunohistochemistry were obtained from lesional and nonlesional skin of 30 patients prior to any treatment. Tissues were cut into 4 *μ*m sections. After deparaffinization and hydration, antigen retrieval was performed and endogenous peroxidase was inactivated with peroxidase blocking solution (Dako, Denmark). Primary antibodies were incubated at the following dilutions: elastin (1 : 100), fibrillin-1 (1 : 50), MMP-2 (1 : 20), MMP-7 (1 : 100), MMP-9 (1 : 20), MMP-12 (1 : 20), TIMP-1 (1 : 20), and TIMP-2 (1 : 50) (Table 1). Primary antibodies were incubated at 4°C overnight. After treatment with secondary antibody, sections were visualized using a DAB kit (EnVisionTM Detection system, Dako, Denmark) and observed under a light microscope. Primary antibodies were replaced with PBS serving as negative controls. The degree of expression was semiquantitatively graded as follows: +, 1–19% positive; ++, 20–79% positive; +++, 80–100% positive. Two independent dermatopathologists scored samples from three high-power fields per section, and the average score was calculated.

### 2.2. Image Analysis

Sections from two patients with protruding lesions and one patient with an indented lesion and their controls and one normal control were analyzed using a computer-based software image analysis program. ImageJ® 1.45 k (Softonic Internacional S.A.) was used to determine the mean optical density for elastogenesis and degradation markers. All mean optical density values were separately measured from the epidermis and dermis. Two serial sections were taken from each paraffin block and image analysis was performed in five fields per tissue section by two independent dermatopathologists.

### 2.3. Statistical Analysis

All values are expressed as mean ± standard deviation (SD). For comparisons between groups,* t*-test and Kruskal-Wallis one-way ANOVA test were conducted. A* p *value of <0.05 was considered statistically significant.

## 3. Results

### 3.1. Demographic Information of Patients with Anetoderma

**Figure 1:**
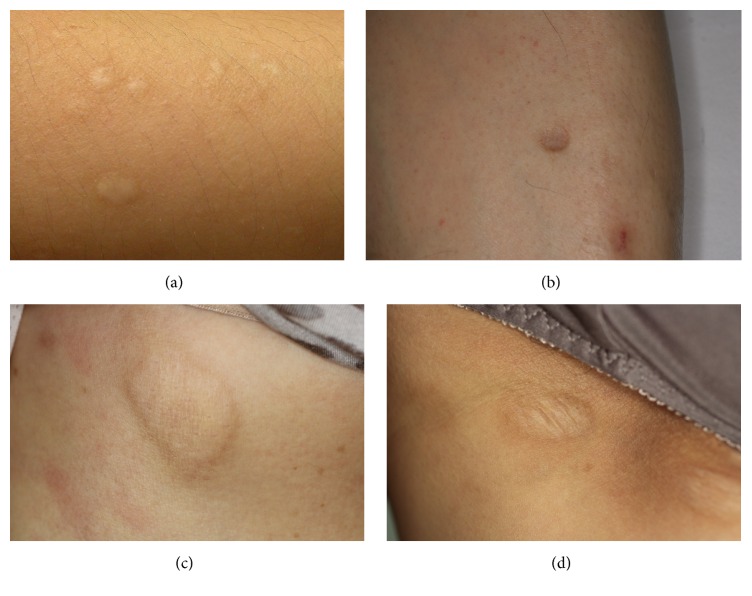
(a) Clinical photo of protruding anetoderma lesions, (b) indented anetoderma lesions, (c) protruding anetoderma lesions of another patient, and (d) indented anetoderma lesions simultaneously found in the same patients who had the protruding lesions shown in (c).

A total of 30 Korean patients (12 males and 18 females) were included in this study. The clinical information of these patients is described in Table 2. Patients with anetoderma were divided into two groups based on their clinical presentation: 17 patients presented with protruding phenotype and 13 patients with indented phenotype. Two patients also presented with a mixed phenotype but had a dominant phenotype (protruding dominant or indented dominant) (Figure 1).

The demographics of the two groups were comparable, and the two groups did not differ in mean age, age of disease onset, number of lesions, or lesion distribution. The age of disease onset ranged from 23 to 37 years (median: 32 years). Disease duration ranged from 3 days to 10 years (median: 12 months). The mean disease duration of patients with the protruding phenotype was 34.3 ± 44.4 months, which was significantly longer than that of patients with the indented phenotype (8.5 ± 10.2 months,* p* < 0.05).

Most patients were in good general health, although some had associated diseases such as hypertension, diabetes mellitus, and chronic hepatitis B infection. No patients had an underlying autoimmune disease. Of the 30 cases in this study, 27 were primary anetoderma. Three patients secondarily developed anetoderma after an antecedent inflammatory event: acne, chicken pox, and hypertrophic scar. Three patients had undergone an antinuclear antibody test. Of those 3 patients, one patient, a 41-year-old woman, showed a positive test result at 1 : 40 dilution with a homogeneous pattern, but she did not develop any rheumatologic symptoms during the follow-up period. We could not check lupus anticoagulant or antiphospholipid antibody in all patients.

The lesions were equally distributed in the face, upper and lower extremities, and trunk. No patients described an association of disease onset with a history of intense UV light exposure.

### 3.2. Hematoxylin and Eosin and Verhoeff-Van Gieson Stains

There was no epidermal change from the lesions, and the thickness of dermal collagen was similar to that of the controls. Elastic fibers were completely or partially decreased from the papillary dermis to the reticular dermis in all anetoderma lesions. The duration of cases showing inflammatory cell infiltrates ranged from 3 days to 2 years (median: 2.5 months). Mild to moderate perivascular lymphohistiocytic inflammatory cell infiltrates were observed in 75% of the early (<12 months) lesions. Scant inflammatory infiltrates were also seen in 44.4% of the late (≥12 months) lesions. The prevalence of inflammatory infiltrates was similar between protruding (7/17, 41.2%) and indented (5/10, 50%) phenotypes. The prevalence of inflammatory infiltrates in long-standing lesions over 12 months was 44.4% (4/9) and 40% (2/5) in protruding and indented lesions, respectively. No vascular changes or microthrombi in vessels were detected in any of the specimens.

### 3.3. Immunohistochemistry

#### 3.3.1. Elastin and Fibrillin-1

**Figure 2:**
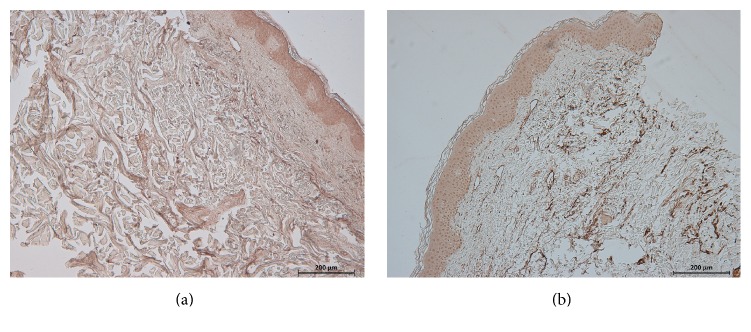
The degree of destruction of elastic fibers was greater in the protruding type than the indented type. (a) Protruding anetoderma lesions; (b) indented anetoderma lesions (elastin, original magnification: ×100).

Elastin immunoreactivity completely or partially decreased from the papillary dermis to the reticular dermis in all anetoderma lesions compared to controls. Elastic fiber destruction was evenly distributed throughout the dermis, with the exception of 4 cases: two cases with protruding lesions and 1 case with indented lesion showed decreased elastic tissue from the mid-dermis to the deep dermis. In contrast, the fourth patient, who had an indented lesion, showed decreased elastic tissue only in the upper dermis. The pattern of elastin destruction did not differ between the protruding type and the indented type. Interestingly, the degree of elastic fiber destruction was greater in the protruding type than the indented type (Figure 2, Table 3). Severe to total loss of elastin was seen in 66.6% of protruding type lesions and 40% of indented type lesions (Table 3). Patients with protruding type lesions had significantly longer disease duration than those with indented type lesions.

Fibrillin-1 was immunopositive in the epidermis and dermal vasculature but was difficult to detect in the dermis of anetoderma lesions as well as controls with the unaided eye.

#### 3.3.2. MMP-2, MMP-9, MMP-7, and MMP-12

MMP-2 and MMP-9 were diffusely expressed in epidermal keratinocytes and occasionally in the cytoplasm of spindle cells located in the lesioned dermis. Diffuse epidermal expression of MMP-2 and MMP-9 was also observed in uninvolved skin and in healthy controls. However, dermal expression of MMP-2 and MMP-9 was not observed in uninvolved skin or healthy controls. MMP-7 and MMP-12 showed little or no expression in lesional and nonlesional skin, as well as in normal controls.

**Figure 3:**
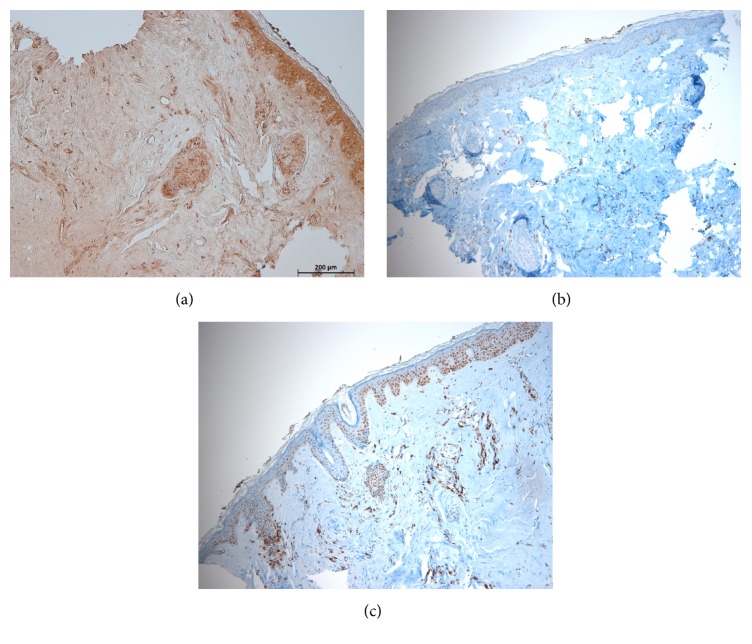
(a) Most MMP-9-expressing spindle shape cells in the dermis showed immunopositivity for (b) CD-68 or (c) CD45 (magnification: ×100).

MMP-9 intensely stained the cytoplasm of spindle-shaped cells in the lesional dermis in several (*N* = 12) patients. From these cases, specimens from three protruding lesions and specimen from one indented lesion were stained with CD45, CD68, and CD1a to investigate the origin of these spindle-shaped cells. The spindle-shaped cells were found to be CD45- or CD68-positive lymphohistiocytes in the specimens of anetoderma patients. There were no MMP-9-expressing reactive fibroblasts (Figure 3). Enhanced MMP-2 expression in dermal fibroblasts was mostly seen in specimens with inflammatory infiltrates.

Among the 12 early (<12 month) anetoderma lesions, 6 (50%) had dermal inflammatory infiltration and those cells showed positive immunoreactivity for MMP-9. Six of the 18 late (>12 month) anetoderma lesions (33.3%) showed positive MMP-9 expression in the lymphohistiocytes in the dermis. Among indented type lesions, 80% and 50% showed positive MMP-2 and MMP-9 expression in the dermis, respectively. In contrast, protruding type lesions showed positive MMP-2 and MMP-9 expression in 29.4% and 41.2% of cases, respectively (Table 4).

#### 3.3.3. TIMP-1 and TIMP-2

**Figure 4:**
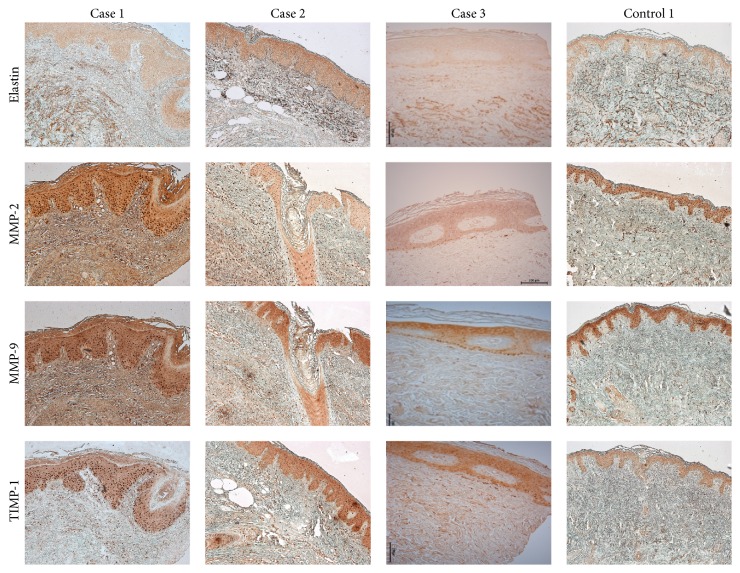
Immunohistochemical study results for elastogenesis and degradation markers in protruding and indented type anetoderma (cases 1 and 2: protruding anetoderma lesions, case 3: indented anetoderma lesion, and control 1: control of case 1) (magnification: ×100).

TIMP-1 was diffusely stained in the epidermal keratinocytes and occasionally stained in a few spindle cells in the dermis in most specimens of anetoderma patients and healthy controls (Table 3 and Figure 4). In contrast, TIMP-2 was variably stained in epidermal keratinocytes and some spindle cells in both anetoderma lesions and controls.

### 3.4. Image Analysis

**Figure 5:**
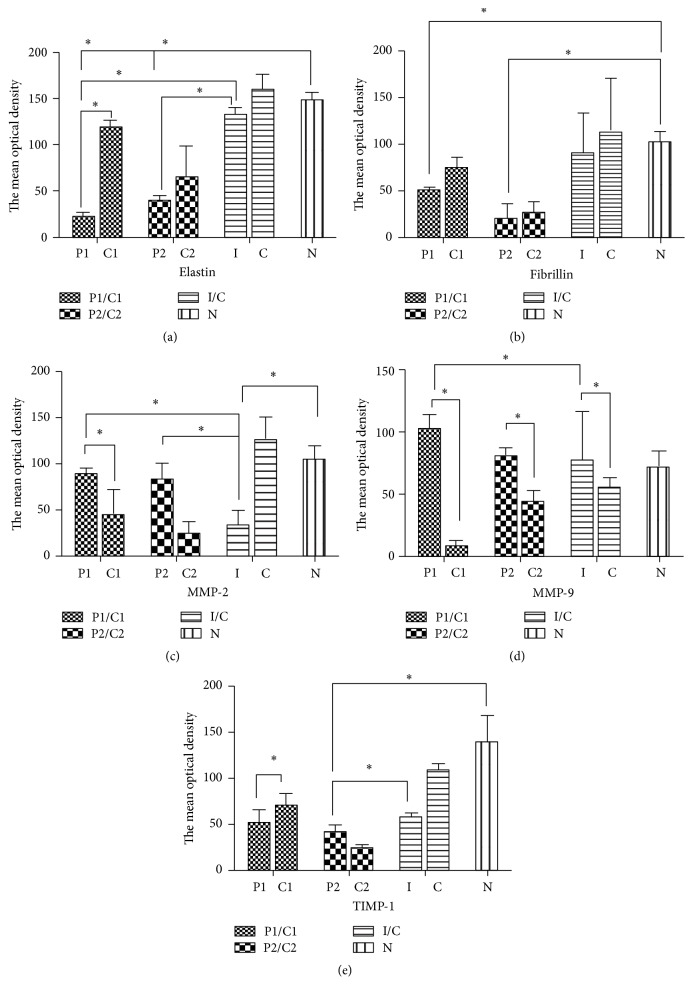
The mean optical density for elastogenesis and degradation markers in the dermis of protruding and indented type anetoderma. (a) Elastin, (b) fibrillin, (c) MMP-2, (d) MMP-9, and (e) TIMP-1 (P1: protruding lesion from case 1, C1: nonlesional control from case 1, P2: protruding lesion from case 2, C2: nonlesional control from case 2, I: indented lesion from case 3, C: nonlesional control from case 3, and N: control from normal patient) ^*∗*^*p* < 0.05:* t*-test, between two groups.

The mean optical density for elastin was available in 3 anetoderma lesions (2 protruding lesions and 1 indented lesion) and corresponding uninvolved control tissue. The mean optical density for elastin from the protruding lesions was significantly lower compared with the controls and indented lesions and normal controls (*p* < 0.05) (Figures 4 and 5, Table 5). The mean optical density for fibrillin-1 was significantly lower in protruding lesions than in corresponding controls. However, there was no difference in the mean optical density of fibrillin-1 expression between indented lesions and their corresponding controls (Table 5).

Both MMP-2 and MMP-9 epidermal expression, assessed by mean optical density, were not significantly different between lesions and uninvolved skin in 3 anetoderma patients. However, the mean optical density for MMP-9 in the dermis was consistently higher in all anetoderma lesions regardless of clinical phenotype compared with uninvolved control tissue, as well as with skin from healthy control patients (*p* < 0.05). The results of the mean optical density values for MMP-2 in the dermis were similar to that of MMP-9; however, values from indented lesions did not differ significantly from those of normal controls (Figures 4 and 5, Table 5).

In terms of TIMP-1 expression in the epidermis and dermis, there was no difference in the mean optical density between anetoderma lesions and controls (Figures 4 and 5, Table 5). For uninvolved skin of patients with anetoderma, the mean optical density of MMPs/TIMPs was not significantly different from that of normal controls.

## 4. Discussion

We examined the clinical and histopathological correlations of different types of anetoderma. Anetoderma lesions showed a variable pattern of loss and fragmentation of elastin in the dermis depending on the stage and the severity of the disease. In this study, several pieces of evidence suggested that the protruding type may be a more advanced stage and severe form of anetoderma. First, patients with protruding type lesions showed a longer disease duration and more severe loss of elastin than patients with indented type lesions. Second, among patients with inflammatory infiltrates, those with protruding type lesions showed more intense MMP-2 and MMP-9 expression than those with indented type lesions. The mean optical density values of MMP-2 and MMP-9 were also higher in protruding type lesions than in indented type lesions. Third, two patients presented with multiple lesions showing both indented and protruding type, which might suggest an evolution of indented lesions into protruding lesions. Certain patients with anetoderma seem to reach a chronic subclinical inflammatory stage and undergo progressive elastic tissue degradation until it ultimately changes to the protruding feature. From these findings, one could speculate that protruding type is a more advanced stage of anetoderma and destruction of elastic fibers by MMP-2 and MMP-9 could be the main pathogenesis of anetoderma.

Elastin was the main target of elastic tissue degradation in our results. Consistent with this observation, a previous study examined electromicroscopy findings of anetoderma and reported that anetoderma is a condition leading to loss of elastin and relative conservation of the microfibrils [17]. Interestingly, the mean optical densities of elastin and fibrillin in our study suggest that protruding type anetoderma may result from both elastin and fibrillin defects, while the indented type may arise from elastin defects only. In patients with anetoderma who have severe destruction of elastic tissue, even loss of fibrillin may be possible, although we could not confirm this result with ultrastructural studies.

MMP-2 and MMP-9 are gelatinases A and B. In humans, MMP-9 has a greater capacity to degrade elastin than MMP-2. MMP-2 is constitutively expressed by keratinocytes and fibroblasts [16]. Enhanced MMP-2 expression in dermal fibroblasts was mostly seen in specimens with inflammatory infiltrates in this study and thus seems to be activated by surrounding inflammatory cytokines. MMP-9 is produced primarily by keratinocytes, macrophages, and fibroblasts [16]. In this study, MMP-9-expressing large spindle cells scattered in the dermis and perivascular areas were mostly CD45- or CD68-positive, suggesting a lymphohistiocyte origin. Thus, lymphohistiocyte-derived elastases may be the major source of degradation of elastic tissue in anetoderma.

Previous studies have reported increased activity of MMP-2 and MMP-9 in skin explant cultured tissue of patients with inflammatory anetoderma [15]. Consistent with this finding, MMP-2 and MMP-9 seemed to contribute to degradation of elastic fibers in our study. However, others have reported anetoderma arising from marginal zone B cell lymphoma in which MMP-9, but not MMP-2 or MMP-12, is expressed in infiltrating lymphocytes, suggesting some degree of pathogenic heterogeneity for this disease [18].

Mid-dermal elastolysis (MDE), which is similar to anetoderma but presents clinically as fine wrinkles, histopathologically shows selective degradation of elastin only in the mid-dermis [19]. Patroi et al. reported that MMP-2 and MMP-9 are increased in MDE lesions. However, they reported that the MMP-9-expressing fibrohistiocytes-like cells in these lesions were reactive fibroblasts, not lymphohistiocytes [20]. While both MDE and anetoderma are considered acquired elastolytic disorders [1], both diseases have different cellular origins leading to the degradation of elastic tissue. Reactive fibroblasts seem to contribute to an MMP-2 rich dermal microenviroment and do not seem to involve MMP-9 secretion in anetoderma. Recently, Gambichler et al. observed decreased fibulin-4 expression in patients with anetoderma or MDE, whereas decreased fibulin-5 expression was only observed in patients with MDE [21].

Cutis laxa encompasses a heterogeneous disease group characterized clinically as loose and redundant skin and histopathologically as loss or fragmentation of elastic fibers. Unlike anetoderma and MDE, cutis laxa often involves internal organs. Several gene defects, such as those in elastin or fibulin 5, have been found in cutis laxa, suggesting an etiology of defects in elastic fiber synthesis. However, there is also evidence that supports the hypothesis that cutis laxa can arise from an imbalance between MMPs and TIMP. Levels of dermal expression of MMP-3, MMP-9, and MMP-12 are increased in cutis laxa lesional fibroblasts or lymphocytes and are correlated with the degree of disruption of elastic fibers [22]. In this study, the degree of elastic tissue degradation was higher in anetoderma patients with stronger immunointensity for MMP-2 and MMP-9 and longer disease duration but was not exactly proportionate since the degradation process of elastin is dynamic.

MMP-7 (matrilysin-1) can bind to fibrillin and cleave fibulin [16]. MMP-7 and MMP-12 (stromelysin, macrophage elastase) showed little or no expression in all specimens of our patients. Consistently, Vaalamo et al. did not detect either MMP-7 or MMP-12 expression in anetoderma tissue by in situ hybridization and immunohistochemistry [23]. However, Ghomrasseni et al. reported that MMP-7 and MMP-12 are involved in the degradation of elastic tissue in anetoderma through organ culture studies from skin explants [14]. This discrepancy may result from the variation of patient characteristics such as clinical phenotype or duration of the lesions and methodological differences.

TIMP-1 seems to be the most effective endogenous inhibitor, binding to activated interstitial collagenase and gelatinases [16]. TIMP-1 was moderately to strongly immunopositive in most anetoderma lesions, but also in controls. Thus, elevated TIMP levels seem insufficient to suppress the highly increased level of MMPs. In contrast to our findings, Venencie et al. reported upregulation of MMP-2 and MMP-9 and downregulation of TIMP-1 in anetoderma lesions [15]. TIMP-2 is known to selectively regulate MMP-2 activity [16]. Thus, little to no expression of TIMP-2 in the setting of significantly elevated MMP-2 in anetoderma skin contributes to imbalance of these enzymes.

Since the number of patients in each clinical phenotype was small, further studies with larger sample sizes are needed to validate and clarify our findings.

We provide various clinical and histopathologic characteristics of anetoderma with a wide range of disease duration and clinical phenotypes. Our findings suggest that the protruding sac phenotype may represent a more advanced stage of anetoderma and that MMP-2 and MMP-9 could be key players in the elastolysis seen in anetoderma [24].

## Figures and Tables

**Table 1:** Primary antibodies used in the study.

Specificity	Reactivity	Source
Elastin	Elastin	Leica
Fibrillin-1	Fibrillin-1	Abcam
MMP-2	MMP-2	R&D Systems
MMP-7	MMP-7	Abcam
MMP-9	MMP-9	R&D Systems
MMP-12	MMP-12	R&D Systems
TIMP-1	TIMP-1	R&D Systems
TIMP-2	TIMP-2	Abcam

MMP: metalloproteinase; TIMP: tissue inhibitor of metalloproteinase.

**Table 2:** Demographic information of 30 anetoderma patients according to clinical phenotypes.

	Protruding type (*n* = 17)	Indented type (*n* = 13)
Sex, M : F	7 : 10	5 : 8
Age (years)	24.1 ± 14.2	33.5 ± 21.4
Disease duration (months)	34.3 ± 44.4 (median: 48)	8.5 ± 10.2 (median: 12)
Affected areas	Face: 4	Face: 3
	Neck: 1	Upper extremities: 4
	Upper extremities: 3	Lower extremities: 4
	Trunk: 11	Trunk: 6
Number of lesions	Solitary lesion: 13	Solitary lesion: 3
	Multiple lesions: 4	Multiple lesions: 10
Previous skin disease	2 (hypertrophic scar, varicella)	0

Variables are shown as mean ± SD.

**Table 3:** Immunohistochemistry results for elastogenesis and degradation markers in anetoderma lesional dermis according to clinical phenotype.

	Intensity of immunostaining				
	0	+	++	+++	total
Elastin					
Protruding type	2	10	5	0	17
Indented type	1	3	6	0	10
Control	0	0	5	0	5
Fibrillin					
Protruding type	8	9	0	0	17
Indented type	1	8	0	0	9
Control	0	1	4	0	5
MMP2					
Protruding type	12	2	3	0	17
Indented type	2	5	3	0	10
Control	3	2	0	0	5
MMP9					
Protruding type	10	3	4	0	17
Indented type	5	2	3	0	10
Control	5	0	0	0	5
TIMP1					
Protruding type	10	2	5	0	17
Indented type	5	3	2	0	10
Control	0	2	3	0	5
TIMP2					
Protruding type	9	0	1	0	10
Indented type	7	2	0	0	9
Control	4	1	0	0	5

The degree of expression was graded as follows: 0, 0% positive; +, 1–19% positive; ++, 20–79% positive; +++, 80–100% positive.

**Table 4:** Immunohistochemistry results for MMP-2 and MMP-9 in anetoderma lesional dermis according to clinical phenotype and disease duration.

	Indented			Protruding		
	0	+	++	0	+	++
MMP-2						
<1 month	0	1	0	1	0	2
1–12 months	0	2	2	4	1	0
>12 months	2	2	1	7	1	1
MMP-9						
<1 month	1	0	0	1	1	1
1–12 months	1	1	2	4	1	0
>12 months	3	1	1	5	1	3

**Table 5:** Mean optical density of elastogenesis and degradation markers in anetoderma.

Group	Disease duration	Epidermis					Dermis				
		Elastin	Fibrillin	MMP-2	MMP-9	TIMP-1	Elastin	Fibrillin	MMP-2	MMP-9	TIMP-1
Case 1, (protruding lesion)	12 months	0.00	71.87 ± 4.16	130.96 ± 13.05	104.23 ± 4.88	103.10 ± 12.71	22.54 ± 4.47	50.91 ± 3.29	88.19 ± 6.66	102.66 ± 11.57	51.75 ± 14.67
Control 1	NA	0.00	108.30 ± 19.12	125.98 ± 6.45	103.40 ± 6.06	81.71 ± 2.07	119.46 ± 7.15	74.32 ± 10.73	45.30 ± 25.72	8.63 ± 7.66	70.37 ± 13.62
Case 2, (protruding lesion)	1 week	0.00	56.16 ± 8.75	62.39 ± 10.45	107.69 ± 8.14	100.27 ± 6.46	39.62 ± 5.21	19.67 ± 15.86	83.16 ± 16.92	80.49 ± 7.18	42.00 ± 8.08
Control 2	NA	0.00	56.68 ± 10.56	44.45 ± 25.07	100.70 ± 5.78	49.29 ± 6.89	65.13 ± 33.51	25.72 ± 13.25	23.26 ± 13.42	43.88 ± 8.85	25.10 ± 3.25
Case 3, (indented lesion)	18.7 months	0.00	88.86 ± 15.5	72.08 ± 5.55	100.79 ± 7.16	95.22 ± 15.34	132.99 ± 7.19	89.33 ± 43.52	32.73 ± 16.18	77.28 ± 39.22	58.03 ± 4.65
Control 3	NA	0.00	99.68 ± 2.81	98.22 ± 4.99	53.35 ± 18.21	114.52 ± 16.75	159.44 ± 16.87	112.91 ± 57.10	125.58 ± 24.57	55.27 ± 8.08	109.37 ± 6.62
Normal control	NA	0.00	124.89 ± 5.90	92.93 ± 9.45	29.29 ± 3.29	108.33 ± 11.59	148.47 ± 8.33	103.03 ± 10.37	104.82 ± 13.92	72.08 ± 12.39	139.56 ± 28.57

NA: not applicable.
